# Hobnail variant of papillary thyroid carcinoma with anaplastic dedifferentiation co-existent with tuberculosis lymphadenitis

**DOI:** 10.1016/j.ijscr.2021.106690

**Published:** 2021-12-18

**Authors:** Abdulwahid M. Salh, Fahmi H. Kakamad, Shko H. Hassan, Ari M. Abdullah, Mohamad A. Hassan, Berwn A. Abdulla

**Affiliations:** a Smart Health Tower, François Mitterrand Street, Sulaimani, Kurdistan, Iraq; b College of Medicine, University of Suleiman, Madam Mettarrand Street, Sulaimani, Kurdistan, Iraq; c Kscien Organization, Hamdi Str., Azadi Mall, Sulaimani, Kurdistan, Iraq; d Shorsh Teaching Hospital, Sulaimani, Kurdistan, Iraq

**Keywords:** Papillary thyroid carcinoma, Hobnail variant, Anaplastic thyroid carcinoma, Tuberculosis

## Abstract

**Introduction:**

The current study aims to report a rare case of metastatic papillary thyroid carcinoma (PTC) of the cervical lymph nodes with hobnail variant and anaplastic de-differentiation. In addition to the primary disease, there was a second pathology which was caseating granulomatous lymph adenitis suggestive of tuberculosis.

**Case report:**

A 91-year-old female presented with a painful right sided neck swelling for two weeks, increased in size suddenly. On clinical examination, there was a well-defined firm painful right sided neck mass. On ultrasound examination, there was multiple well defined solid hypoechoic, hypervascular nodules. These resembled lymph nodes of variable size and shape, mostly in the right side. The patient underwent right lateral cervical lymph node dissection. After the operation, she was sent for radiotherapy.

**Discussion:**

The hobnail variant of PTC is genetically identical to poorly differentiated thyroid carcinoma in that its mutations are in the p53 and TERT promoters are more common in this variant than in conventional PTCs. The proportion of hobnail features have no effect on the outcome. Additionally, 10% of tumor cells with hobnail features were previously linked to a more aggressive clinicopathological aspect.

**Conclusion:**

Although it is rare, metastatic PTC with hobnail variant could undergo anaplastic dedifferentiation.

## Highlights

• Thyroid carcinoma accounts for nearly 1% of all malignancies.
• The hobnail variant of papillary thyroid carcinoma (HPTC) is a rare condition.
• Anaplastic thyroid carcinoma (ATC) is a rare form of thyroid cancer.
• Malignant lesions and tuberculosis coexisting at the same site is exceedingly unusual.

## 1 Introduction

Thyroid carcinoma accounts for 1% of all malignancies and 0.2% of all cancer deaths. The majority of these tumors are papillary in nature. In countries with iodine-sufficient or iodine-excess diets, this is the most frequent malignant tumor of the thyroid gland, accounting for 80–85% of thyroid malignancies [1]. Papillary thyroid carcinoma (PTC) is the commonest endocrine malignancy, with thyroid cancer being the fifth most common malignancy in female and the fastest growing cancer in both sexes in the United States during the last decade [2]. Several variants of PTC have been identified and may be related with more aggressive clinical behavior [3]. PTC variants account for up to 25% of all cases. Size, tumor boundaries, architecture, cellular characteristics, stromal features, or a combination of the above-mentioned criteria have been used to describe the variants [4]. The hobnail variant of PTC (HPTC) is an extremely uncommon condition [5].

Anaplastic thyroid carcinoma (ATC) is a rare and the most aggressive form of thyroid cancer accounting for nearly 1.7% of all thyroid cancers in the USA [6]. Although, it is assumed that ATC is usually caused by the dedifferentiation of well differentiated thyroid cancer (WDTC), the clinicopathological characteristics of this dedifferentiation is poorly understood [7]. Malignancies, whether primary or metastatic, and infections are the most common causes of cervical lymphadenopathy and should be included in the differential diagnosis [8]. Tuberculosis (TB) is still one of the world's deadliest illnesses and the top cause of death due to infectious disease [9]. Malignant lesions and tuberculosis coexisting at the same anatomical site in a patient is exceedingly unusual [10].

The current study aims to report an extremely rare case of metastatic PTC of the cervical lymph nodes with hobnail variant and anaplastic dedifferentiation, as well as caseating granulomatous lymph adenitis, which suggested TB. The report has been arranged in line with SCARE 2020 guidelines with a brief literature review [11].

## 2 Case report

### 2.1 Patient information

A-91-year-old age female presented with a painful right-sided neck swelling for two weeks that had suddenly increased in size. She had previous surgical history of eye surgery and uterine operation a long time ago. She had thyroid cancer and underwent thyroidectomy in 2010, after this operation she received radioactive iodine. She had a past medical history of hypertension, on anti-hypertensive medication and thyroxine table (100 microgram) once per day since her operation.

### 2.2 Clinical findings

On clinical examination there was significant voice change with well-defined firm tender right sided neck mass that was neither ulcerated or red in the skin over the swelling. On vocal cord examination, there was right vocal cord paralysis with a normal left vocal cord.

### 2.3 Diagnostic assessment

The patient's TSH was (<0.05uUI/), ESR (52 mm/h), and S. Calcium (9.81 mg/dl). Ultrasound of the neck revealed a well-defined solid (mildly vascular) nodule (16*15*17 mm) in the lower portion of the right operation bed, most likely a suspicious lymph node. Multiple well-defined solid hypoechoic hyper-vascular nodules of lymph nodes of varying sizes were detected in the right anterior triangle of the neck, the largest one was (55*49*30 mm) in the right groups III and IV, and the second largest one was (20*15 mm) in the right group II. There were no focal lesions in the left operation bed. 2–3 tiny lymph nodes were found in groups III and IV on the left side but they had no pathological futures; the largest one was (9*4 mm). There were no localized lesions in the submandibular and parotid glands.

A CT of the neck, chest, abdomen, and pelvis was performed with IV contrast and revealed a 22 mm mass lesion with complement encasement of the right common carotid artery, another 11 mm oval shaped, most likely a pathological lymph node, in the parapharyngeal space and multiple smaller lymph nodes in both sides of the neck and right paratracheal region. There was also a single small 4 mm nodule in the right upper lobe of the lung. The FNAC was taken from the right cervical lymph node, and the outcome showed metastatic carcinoma with a tendency favoring thyroid cancer origin.

### 2.4 Therapeutic intervention

**Fig. 1:**
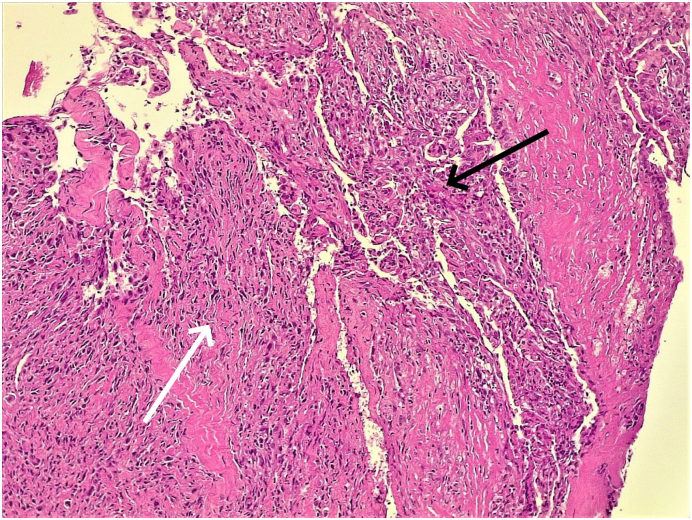
Section of an involved cervical lymph node showing the interface between an area of conventional papillary thyroid carcinoma (black arrow) with remnants of papillary structures and nuclear features, and an area of dedifferentiation towards anaplastic carcinoma (white arrow) with higher-grade nuclear atypia and sheet-like architecture.

The patient underwent right lateral cervical lymph node dissection, and the surgical specimen was sent for histopathological examination (HPE). HPE revealed metastatic PTC with a hobnail variant and anaplastic dedifferentiation, as well as caseating granulomatous adenitis, which is indicative of tuberculosis (Fig. 1). 6 out of 36 lymph nodes were involved with extra nodal extension of PTC and pathological stage of pTx N1b was determined.

### 2.5 Follow up

After the operation, she was sent for radiotherapy and she took five sessions of radiotherapy which caused her severe weakness.

## 3 Discussion

Well differentiated thyroid cancer is the most common endocrine malignancy, its incidence ratio has continuously increased in the recent decades, with the papillary histotype being the most common [12]. There are multiple histological variants of PTC, which exhibit distinct growth patterns, cell types, stromal alterations, and genetic mutations. According to the recent reports, the HPTC, also known as the micropapillary variant, is a rare variant accounting for 0.2% to 0.3% of PTCs [13], [14]. According to the WHO criteria, a tumor must contain at least 30% of hobnail cells to be classified as hobnail variant [15]. When compared to primary PTC, the percentage of hobnail component appeared to rise in metastases [16]. It is a moderately differentiated variant with aggressive clinical characteristics and high mortality [17]. The HPTC is genetically identical to poorly differentiated thyroid carcinoma (PDTC) in that those mutations in the p53 and TERT promoters are more common in this variant than in the conventional PTC [12]. The proportion of hobnail features have no effect on the outcome. Even 10% of tumor cells with hobnail features were previously linked to an aggressive clinicopathological aspect [18]. A multistep process of genetic and epigenetic alterations can cause well-differentiated thyroid carcinomas to dedifferentiate, resulting in a poorly differentiated or undifferentiated/ATC [19].

ATC is the most aggressive form of thyroid neoplasm that may develop de novo or from a pre-existing WDTC [20], [21]. Although, the transformation of PTC to more aggressive ATC in the primary thyroid site is a well-known occurrence, the transformation of metastatic PTC in cervical lymph nodes is an extremely rare finding [22]. Tuberculous adenitis is a prevalent disease in several regions of the world. TB can be classified into pulmonary and extrapulmonary TB. Extrapulmonary TB accounts for 20% to 30% of all tuberculosis cases, with tuberculosis in the head and neck areas accounting for 10% [9]. The prevalence of TB has been observed to be higher than that of PTC-related metastatic deposits in the cervical lymph nodes [8]. Without pulmonary or thyroid TB, the presence of tuberculosis with metastatic cancer is a rare condition [23].

The pathophysiology behind the hobnail variant's aggressive behavior is not well understood. Previous researches have shown that the hobnail pattern might be a sign of epithelial-mesenchymal transition (EMT). EMT is a well-known pathological process in epithelial tumor development, local invasion, and metastasis that has been linked to the HPTC, as well as PDTC and ATC [24]. The method of anaplastic transition in WDTCs are yet unknown. Over-'[-expression of p53 and Ki-67 might be linked with anaplastic transformation [25]. A significant proportion of patients with PTC receive radioiodine treatment. The relationship between radioiodine and anaplastic transformation has been investigated in a few animals and cell lines studies, and it has been shown to be minimal. [26]. The most common mode of presentation is as thyroid nodule or cervical lymphadenopathy. Although incidental detection of non-palpable papillary thyroid carcinoma is common, distant metastasis as a presenting feature is a rare occurrence [27]. Because synchronous simultaneous incidence of TB and metastatic carcinoma is uncommon, it might be challenging for the clinicians [23]. Cervical TB lymphadenitis can manifest as a single or numerous painless masses in the supraclavicular region or the posterior triangle of the neck [28]. The current case presented eleven years after the primary operation with a painful swelling in the right side of the neck.

Ito et al. reported five patients with primary thyroid PTC who developed anaplastic transformation in cervical lymph nodes. The main thyroid lesion was 0.4 to 3.5 cm in diameter. The anaplastic metastases were discovered in two individuals during the original, main surgical operation. The other three patients presented after four, six, and 20 years respectively following the initial surgical procedure. After six months and seven years of follow-up, the two patients who were treated during the main operation for their cervical anaplastic metastases were still alive. Patients who underwent a late anaplastic transition died of cancer. It is worth noting that none of the patients received radioiodine therapy [29].

Hobnail cells vary in size and form from tiny lymphocytoid cells to larger cuboidal cells and to tall/columnar cells in severe cases. A hobnail pattern has been observed in a variety of malignancies from various organs, including the ovaries, where it has been linked to both benign and malignant tumors [30]. This variant is distinguished histologically and cytologically by three distinct architectural and cytologic features: micro-papillae lacking true fibrovascular cores, cells with eosinophilic cytoplasm and apically placed nuclei with a decreased nucleus/cytoplasm ratio (hobnail appearance), and loss of cellular cohesion that form characteristic hobnail cells in at least 30% of the neoplastic cells [17], [31]. In recurrent PTCs, an increased mitotic count, the appearance of necrosis, high grade features, might be predictors of a subsequent anaplastic transition [32]. Most typical PTCs do not display solid and insular development, the appearance of these components in recurrent PTCs might indicate an anaplastic transition [32]. Amacher et al. reported when hobnail pattern is observed in PTC, it is strongly associated with other histologic variants known to be more clinically aggressive [16].

Cancer recurrence or metastasis to lymph nodes and/or distant organs occurs in around 10% of PTC patients [30]. Even in the metastatic situation, PTC is linked with relatively excellent survival, tumor-related death and accounts for less than 5% in most studies [33]. However, some clinical and pathological characteristics of PTC have been linked to an increased risk of tumor recurrence and cancer-related death. Age at diagnosis, the size of the original tumor, and any soft tissue invasion or distant metastases are the most critical variables. Some PTC subtypes, such as tall cell and hobnail variants, are also linked to a poor prognosis [12]. Recent reports revealed extrathyroidal extension in more than half and lymph node metastases in nearly 70% of the HPTC cases [13]. When hobnail variant even presents in as little as 5% of the tumor, it is a predictor of lymph node metastases and causes cancer-related mortality more frequently than the typical form of PTC. After 8.5 years of follow-up, mortality has been reported to be as high as 57%. This mortality rate is significantly greater than the 10% for typical PTC [16]. Anaplastic transition is a critical event in the prognosis of differentiated carcinoma [29]. ATC has a bad prognosis, with a median survival rate of 4 to 12 months and a 5-year survival rate of 1.0% to 7.1% [22].

In conclusion, although it is rare, metastatic HPTC co-existent with TB could undergo anaplastic dedifferentiation. When compared to conventional PTC, HPTC is significantly associated with aggressive clinicopathologic characteristics, disease progression, greater mortality rate and has a potential to undergo anaplastic transition.

## Sources of funding

None is found.

## Ethical approval

Not required for case report.

## Consent

Written informed consent was obtained from the patient for publication of this case report and accompanying images. A copy of the written consent is available for review by the Editor-in-Chief of this journal on request.

## Guarantor

Fahmi Hussein Kakamad

Fahmi.hussein@univsul.edu.iq

## CRediT authorship contribution statement

Abdulwahid M. Salh: surgeon performing the operation, major contribution of the idea, literature review, final approval of the manuscript.

Fahmi H. Kakamad: Surgeons performing the operation, final approval of the manuscript.

Shko H. Hassan, Ari M. Abdullah, Mohamad A. Hassan: literature review, final approval of the manuscript.

Berwn A. Abdulla: Writing the manuscript, literature review, final approval of the manuscript.

## Declaration of competing interest

None to be declared.
